# Shotgun Metagenomic Survey of the Diseased and Healthy Maize (Zea mays L.) Rhizobiomes

**DOI:** 10.1128/mra.00498-22

**Published:** 2022-09-06

**Authors:** Olubukola Oluranti Babalola, Siphiwe Prudence Dlamini, Akinlolu Olalekan Akanmu

**Affiliations:** a Food Security and Safety Focus Area, Faculty of Natural and Agricultural Sciences, North-West University, Mmabatho, South Africa; Montana State University

## Abstract

The effective functioning of the rhizosphere microbiome significantly contributes to plant development, disease resistance, and agricultural sustainability. Hence, it is a major predictor of plant health. This study evaluated the microbial diversities and functions associated with healthy and diseased maize rhizosphere at selected farms in North West Province, South Africa.

## ANNOUNCEMENT

Despite the contribution of maize to global food security, the prevalence of soilborne diseases significantly reduces its quality and yield (1, 2). In this study, sample collection was carried out from the rhizospheric region of maize plants from Lichtenberg (25°59′40.2″S, 26°31′44.2″E) and Mafikeng (25°47′19.1″S, 25°37′05.1″E), North West Province, South Africa. Each field was demarcated into two areas, which were independently sampled. The respective samples from different healthy and northern corn leaf blight (NCLB)-infected maize plants were pooled into two replicates. The samples were scraped from the soil tightly bound to plant root. In all, four samples were collected from each farm site, and each sample was filtered through a 2-mm sieve, collected in sterile bags, transferred to cooler boxes containing ice packs, and later stored at −20°C in the laboratory until DNA was extracted for shotgun metagenomic sequencing.

A calibrated scale was used to weigh 5 g of stored rhizosphere soil from each sample. The extraction of DNA was carried out using NucleoSpin soil kit (Macherey-Nagel, Germany). The quality of DNA obtained was evaluated using a NanoDrop spectrophotometer.

The libraries were prepared with 50 ng DNA using Nextera DNA Flex kit (Illumina, San Diego, CA, USA; catalog number 20018705), while the samples were subjected to simultaneous fragmentation and addition of adapter sequences. The final concentration of the libraries was measured using the Qubit double-stranded DNA (dsDNA) high-sensitivity (HS) assay kit (Life Technologies), and the average library size was determined using an Agilent 2100 bioanalyzer (Agilent Technologies). The libraries were pooled and diluted to 0.6 nM, and paired-end sequencing was performed for 300 cycles using a NovaSeq system.

The quality control (QC) of raw data, reduction of low-quality reads and removal of replicate data were conducted by SolexaQA v1.6 (3). Duplicate Reads Inferred Sequencing Error Estimation (DRISEE), a package of MG-RAST v4.0.3 (4), was employed to evaluate the error of sequenced samples caused by the artificial replicated sequenced data. To execute the analytical processing downstream and the taxonomic assignment with k-mer, we used the MG-RAST v4.0.3 (4) server with default settings (Table 1).

**TABLE 1:** Sequence reads for the rhizosphere soil samples analyzed^*a*^

Type of data	Sample	LI R1	LI R2	LID R1	LID R2	MA R1	MA R2	MAD R1	MAD R2
Data before QC	Size (bp)	1,615,034,280	896,730,992	37,819,340	1,014,288,894	1,416,428,319	34,607,850	934,504,478	1,275,590,445
	No. of sequence reads	11,021,733	5,814,118	230,808	6,815,631	9,358,543	258,899	6,189,474	9,025,681
	Mean sequence length (bp)	147 ± 70	154 ± 71	164 ± 120	149 ± 72	151 ± 71	134 ± 118	151 ± 72	141 ± 71
	Mean GC content (%)	64 ± 12	64 ± 12	39 ± 34	65 ± 12	64 ± 12	30 ± 34	65 ± 13	65 ± 13
	No. of artificial duplicate read sequences	522,127	261,549	5,657	329,713	470,311	4,650	269,079	446,229
Data after processing	No. of known proteins predicted	8,121,617	4,475,247	119,523	5,054,384	6,897,633	103,708	4,590,770	6,271,956
	No. of RNA features predicted	14,059	9,015	215	9,845	17,342	284	11,588	15,275
Data after QC	Size (bp)	1,490,181,369	841,065,375	32,563,182	942,747,215	1,318,000,396	28,343,946	874,147,383	1,176,234,249
	Sequence count	9,618,671	5,214,826	125,593	5,984,443	8,287,108	110,541	5,481,595	7,735,499
	Mean sequence length (bp)	155 ± 67	161 ± 67	259 ± 61	158 ± 68	159 ± 67	256 ± 64	159 ± 68	152 ± 67
	Mean GC content (%)	65 ± 9	65 ± 9	68 ± 9	66 ± 9	66 ± 9	67 ± 9	66 ± 9	67 ± 8
Data after alignment	No. of known proteins identified	2,910,995	1,657,022	45,046	2,009,779	2,646,340	37,768	1,794,264	2,401,895
	No. of RNA features identified	4,383	3,083	46	3,582	5,086	45	3,863	4,305

a Values are represented as mean ± standard deviation. LA and MA represent rhizosphere soil obtained from healthy maize in Lichtenburg and Mafikeng, respectively. LID and MAD represent rhizosphere soil obtained from diseased maize in Lichtenburg and Mafikeng, respectively.

**FIG 1:**
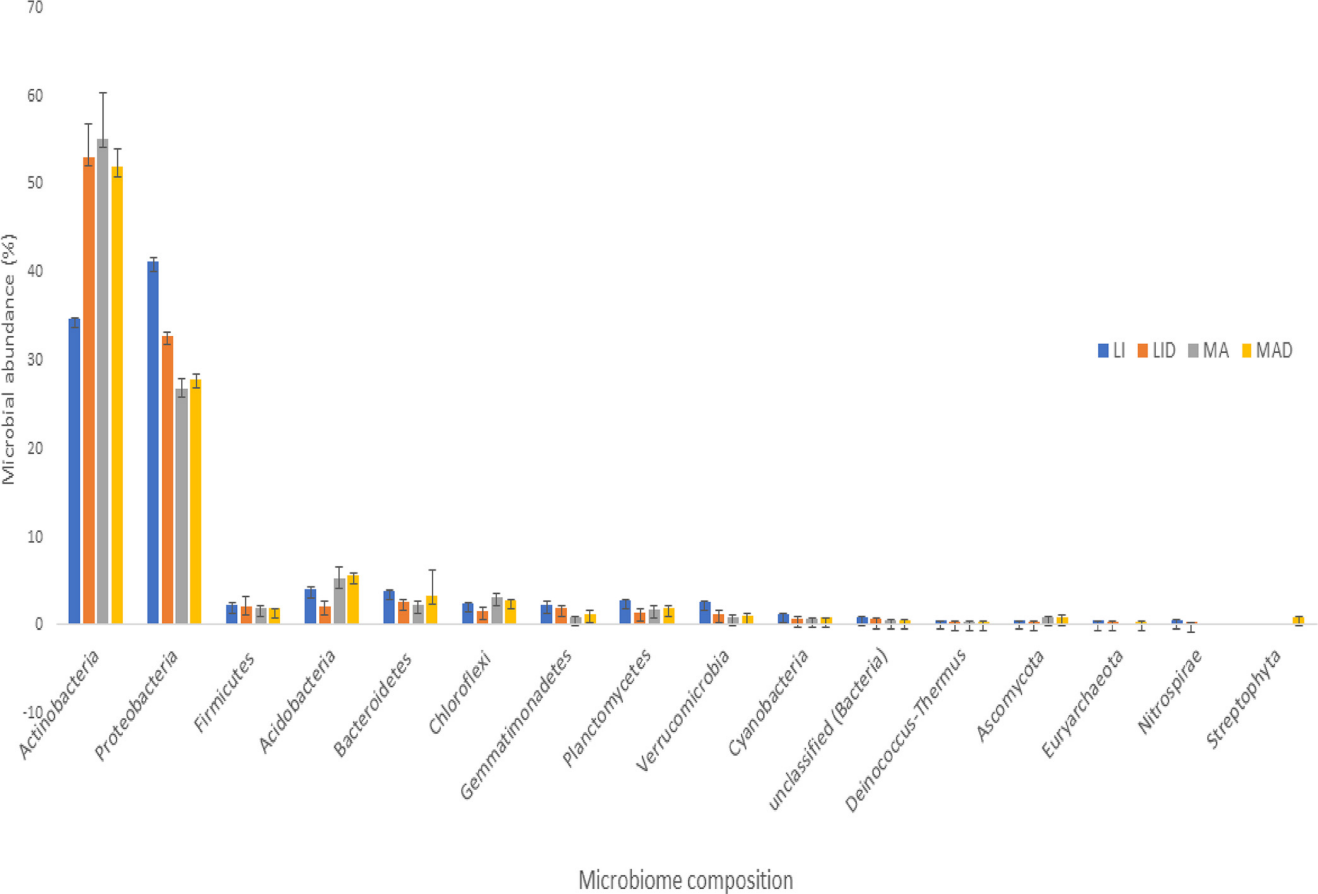
Abundant phyla obtained according to the taxonomic system. Each bar represents the mean ± standard error of each phylum component recovered. LA and MA represent rhizosphere soil obtained from healthy maize in Lichtenburg and Mafikeng, respectively. LID and MAD represent rhizosphere soil obtained from diseased maize in Lichtenburg and Mafikeng, respectively. R, replicate.

*Bacteria*, *Eukaryota*, and *Archaea* are the domains or kingdoms derived from the taxonomy system. The *Bacteria* domain had the most abundant phyla. *Actinobacteria* (34.53 to 55.01%) and *Proteobacteria* (26.6 to 40.98%) were the most abundant, and others, such as *Acidobacteria* (1.95 to 5.52%), *Bacteroidetes* (2.23 to 3.75%), *Firmicutes* (1.75 to 2.21%), *Planctomycetes* (1.29 to 2.71%), *Verrucomicrobia* (0.92 to 2.5%), and *Gemmatimonadetes* (0.82 to 2.14%), were also significant. Additionally, readings for fungi (*Ascomycota*) and archaea (*Euryarchaeota*) were found, albeit at (<1%) relative abundance (Fig. 1).

Functional annotation after mapping with SEED subsystems revealed the essential properties such as carbohydrate-processing enzymes (15.27 to 16.05%); clustering-based systems (13.78 to 13.91%); amino acids and derivatives (11.48 to 11.96%); protein metabolism (6.67 to 6.88%); RNA metabolism (4.41 to 4.61%); fatty acids, lipids, and isoprenoids (4.33 to 4.78%); DNA metabolism (3.71 to 3.84%); cell wall and capsule (3.40 to 3.72%); virulence, disease, and defense (2.26 to 2.51%); and stress response (2.13 to 2.22%), among others.

### Data availability.

The metagenomes of rhizosphere soil sequence reads were submitted to the NCBI under BioProject accession number PRJNA821718 and Sequence Read Archive (SRA) accession numbers SRX14775303 and SRX14775302 (for rhizosphere soil obtained from diseased maize in Mafikeng [MAD]), SRX14775301 and SRX14775300 (for rhizosphere soil obtained from the healthy maize in Mafikeng [MA]), SRX14775299 and SRX14775298 (for rhizosphere soil obtained from diseased maize in Lichtenburg [LID]), and SRX14775297 and SRX14775296 (for rhizosphere soil obtained from the healthy maize in Lichtenburg [LI]).
